# Two Distinct Enzymes Have Both Phytoene Desaturase and 3,4-Desaturase Activities Involved in Carotenoid Biosynthesis by the Extremely Halophilic Archaeon *Haloarcula japonica*

**DOI:** 10.1264/jsme2.ME24004

**Published:** 2024-05-29

**Authors:** Rie Yatsunami, Ai Ando, Nobuhiro Miyoko, Ying Yang, Shinichi Takaichi, Satoshi Nakamura

**Affiliations:** 1 School of Life Science and Technology, Tokyo Institute of Technology, Nagatsuta, Midori-ku, Yokohama 226–8501, Japan; 2 Faculty of Life Sciences, Tokyo University of Agriculture, Sakuragaoka, Setagaya-ku, Tokyo 156–8502, Japan

**Keywords:** carotenoid biosynthesis, phytoene desaturase, 3,4-desaturase, extremely halophilic archaeon *Haloarcula japonica*, bacterioruberin

## Abstract

The extremely halophilic archaeon *Haloarcula japonica* accumulates the C_50_ carotenoid, bacterioruberin (BR). To reveal the BR biosynthetic pathway, unidentified phytoene desaturase candidates were functionally characterized in the present study. Two genes encoding the potential phytoene desaturases, *c0507* and *d1086*, were found from the *Ha. japonica* genome sequence by a homology search using the Basic Local Align Search Tool. Disruption mutants of *c0507* and *d1086* and their complemented strains transformed with expression plasmids for *c0507* and *d1086* were subsequently constructed. High-performance liquid chromatography (HPLC) ana­lyses of carotenoids produced by these strains revealed that C0507 and D1086 were both bifunctional enzymes with the same activities as both phytoene desaturase (CrtI) and 3,4-desaturase (CrtD). C0507 and D1086 complemented each other during BR biosynthesis in *Ha. japonica*. This is the first study to identify two distinct enzymes with both CrtI and CrtD activities in an extremely halophilic archaeon.

Carotenoids, an important class of natural isoprenoid-derived pigments, are synthesized by all photosynthetic organisms and some non-photosynthetic bacteria, archaea, and fungi. They act as light-harvesting pigments in photosynthesis (Holt *et al.*, 2005) and also as antioxidants (Miller *et al.*, 1996; Naguib, 2000) and light-protecting pigments in halophilic archaea (Dundas and Larsen, 1963; Shahmohammadi *et al.*, 1998).

All carotenoids are synthesized from geranylgeranyl pyrophosphate (Takaichi, 2013). In the first step, this compound is formed by geranylgeranyl pyrophosphate synthase (CrtE), which catalyzes the condensation of farnesyl pyrophosphate with an isopentyl pyrophosphate moiety. The second step, catalyzed by phytoene synthase (CrtB), involves the formation of the first C_40_ carotenoid, phytoene from two molecules of geranylgeranyl diphosphate (GGPP). The colorless compound, phytoene, is converted to lycopene through a series of desaturation reactions catalyzed by a bacterial-type phytoene desaturase (CrtI) in bacteria other than cyanobacteria and green-sulfur bacteria (Takaichi and Mochimaru, 2007). Further downstream modification reactions, including the cyclization of lycopene, the addition of keto and/or hydroxy groups, and the introduction of 5-carbon (C_5_) isoprene units, lead to the formation of different carotenoid products. Some carotenoid biosynthetic genes have been characterized in various bacteria and plants (Takaichi, 2013; Sandmann, 2021; Shimada and Takaichi, 2024).

Extremely halophilic archaea forming red-colored colonies produce acyclic C_50_ bacterioruberin (BR) and its precursors (Dummer *et al.*, 2011; Yang *et al.*, 2015). These C_50_ carotenoids increase membrane rigidity (Lazrak *et al.*, 1988) and protect cells against UV light (Shahmohammadi *et al.*, 1998). β-Carotene is the precursor of retinal (Peck *et al.*, 2001). Retinal combines with bacterioopsin to generate bacteriorhodopsin, a light-induced proton pump (Oesterhelt and Stoeckenius, 1971).

The extremely halophilic archaeon *Halobacterium salinarum* produces BR and its precursors, such as iso­pentenyldehydrorhodopin (IDR), bisanhydrobacterioruberin (BABR), and monoanhydrobacterioruberin (MABR), as well as retinal, which constitutes bacteriorhodopsin (Oesterhelt and Stoeckenius, 1971; Dummer *et al.*, 2011). *Hb. salinarum* synthesizes both BR and retinal from a common intermediate, lycopene. In retinal synthesis, lycopene is converted to β-carotene by lycopene β-cyclase (CrtY) (Peck *et al.*, 2002), and β-carotene is then cleaved to form retinal (C_20_) by β-carotene cleavage dioxygenase (Brp) (Peck *et al.*, 2001).

*Haloarcula japonica* is a predominantly triangular disc-shaped extremely halophilic archaeon (Horikoshi *et al.*, 1993). We previously reported that *Ha. japonica* produces some carotenoid, phytoene, lycopene, and BR and its precursors, similar to other extremely halophilic archaea (Yatsunami *et al.*, 2014). In addition, *Ha. japonica* produces the retinal protein, cruxrhodopsin (Yatsunami *et al.*, 1999). We demonstrated that three genes (*c0507*, *c0506*, and *c0505*) encode carotenoid 3,4-desaturase (CrtD), bifunctional lycopene elongase and 1,2-hydratase (LyeJ), and the C_50_ carotenoid 2″,3″-hydratase (CruF), respectively (Fig. 1) (Yang *et al.*, 2015). These three enzymes catalyze the conversion of lycopene to BR in *Ha. japonica*. Based on our previous study and the proposed biosynthetic pathways of BR in *Hb. salinarum*, BR and retinal biosynthetic pathways in *Ha. japonica* are shown in Fig. 2. However, the gene encoding phytoene desaturase in *Ha. japonica* and other extremely halophilic archaea remains unknown.

In the present study, we recharacterized C0507 (previously identified as CrtD) and then characterized D1086, a paralog of C0507. C0507 and D1086 were both found to be bifunctional enzymes, operating as both phytoene desaturases (CrtI) and 3,4-desaturases (CrtD) for BR biosynthesis. The present study is the first functional identification of these novel enzymes in archaea.

## Materials and Methods

### Microbial strains and growth conditions

All strains and plasmids used in this study are listed in Table 1. *Escherichia coli* strains were cultured in LB medium at 37°C (Sambrook *et al.*, 1989). Ampicillin (50‍ ‍μg mL^–1^) was added as required.

Wild-type and genetically modified strains of *Ha. japonica* (JCM 7785^T^) were grown in complex medium at 37°C in the dark, as previously described (Yatsunami *et al.*, 2014). Medium was supplemented with 8‍ ‍μg mL^–1^ of pravastatin (a gift of Daiichi Sankyo), instead of mevinoline, as required.

### Isolation of genomic DNA and total RNA from *Ha. japonica*

*Ha. japonica* genomic DNA was isolated as previously described (Onodera *et al.*, 2013). The total RNA of *Ha. japonica* was extracted using Sepasol RNA I (Nacalai Tesque) according to the manufacturer’s instructions, and was then treated with DNase I (GE Healthcare) to remove any genomic DNA contaminants.

### Reverse transcription-polymerase chain reaction (RT-PCR) and PCR

Oligonucleotide primers for RT-PCR and PCR were purchased from Operon Biotechnologies. All primers are listed in Table 2. RT-PCR was performed as described below. Briefly, total RNA (4.5‍ ‍μg) was reverse-transcribed at 42°C for 60‍ ‍min in 50‍ ‍μL of the reaction buffer, which containing 20 pmol of each primer (c0507-A or d1086-F-A), 0.3‍ ‍mM (each) deoxynucleotide triphosphate, 2.5‍ ‍mM manganese (II) acetate, 20‍ ‍U RNase inhibitor (Toyobo), and 100‍ ‍U reverse transcriptase (ReverTra Ace; Toyobo). The cDNA generated was amplified by 40 cycles of PCR. PCR was performed using KOD-Plus DNA polymerase (Toyobo), according to the manufacturer’s instructions, except for the addition of 10% (v/v) dimethyl sulfoxide. The c0507-S/c0507-A and d1086-F-S/d1086-F-A primer sets were used to confirm the transcription of the *c0507* and *d1086* genes, respectively, in wild-type *Ha. japonica*.

### Plasmids

All plasmids used in this study are also listed in Table 1. pUC119 was used as the cloning vector for *E. coli*. pWL102 is an *E. coli*-extremely halophilic archaea shuttle vector (Lam and Doolittle, 1989). pJFZ33 is a recombinant plasmid containing the *Ha. japonica* cell surface glycoprotein (*csg*) gene promoter sequence, *Nde*I restriction site, and *Ha. japonica ftsZ2* structural gene sequence, *Bam*HI and *Nco*I restriction sites were inserted into pWL102 (Ozawa *et al.*, 2005). *Ha. japonica* contains a large amount of glycoprotein (CSG) on its cell surface, indicating the significance of the *csg* gene promoter. pJ*c0507*, the expression plasmid for the *c0507* gene in *Ha. japonica*, was derived from pJFZ33 in which the *ftsZ2* structural gene was replaced with the *c0507* structural gene, as previously described (Yang *et al.*, 2015).

Part of *d1086* is upstream of the *brp* homolog (*c1158*) in *Ha. japonica* (Yatsunami *et al.*, 2003). Therefore, we cloned the *d1086* gene from the *Pst*I subgenomic library by genome walking using the upstream region of the *c1158* gene as a probe, and termed the plasmid containing the entire *d1086* structural gene as pCRTI2. The *Pst*I-*Hin*cII (containing a part of the *d1086* gene from the start codon to the 474^th^ base) and *Rsa*I-*Pst*I (containing a part of the *d1086* gene from the 1,187^th^ base to the stop codon) fragments of pCRTI2 were ligated to the large *Eco*RI-*Hin*dIII fragment of pWL102 via the linker sequences of pUC119 to construct the recombinant plasmid, pWL102(Δ*ori*)-Δ*d1086*. This plasmid included a disrupted *d1086* gene and lacked the replication origin necessary for replication in extremely halophilic archaea.

The *d1086* structural gene was then amplified from *Ha. japonica* genomic DNA using the primer set, d1086-S3/d1086-A3. The PCR fragment was ligated to the *Sma*I site of pUC119, yielding the pUC119-*d1086* plasmid. The *Nde*I-*Bam*HI fragment of pUC119-*d1086* was ligated to the *Nde*I/*Bam*HI site of pJFZ33 to produce the pJ*d1086* plasmid. This plasmid contained a gene encoding C-terminally His-tagged D1086 under the control of the *csg* gene promoter and was used as an expression plasmid for *d1086* in *Ha. japonica*.

The pWL102(Δ*ori*)-Δ*d1086*, pJ*c0507*, and pJ*d1086* plasmids were initially passed through *E. coli* JM110 to avoid restriction barrier formation in extremely halophilic archaea (Ozawa *et al.*, 2005) and were then transformed in *Ha. japonica*.

### *Ha. japonica* disruptants and their complemented strains

*Ha. japonica* disruptant strains with the deletion mutations were constructed by homologous recombination as previously described (Yang *et al.*, 2015). The disruptant strain, Δ*d1086*, was constructed by transforming pWL102(Δ*ori*)-Δ*d1086* into the *Ha. japonica* wild-type strain. We also used the previously established Δ*c0507* strain (Yang *et al.*, 2015). Additionally, the double-disruptant strain, Δ*c0507*Δ*d1086*, was constructed by transforming pWL102(Δ*ori*)-Δ*d1086* into the Δ*c0507* strain. The disruption of the *d1086* genes in the Δ*d1086* and Δ*c0507*Δ*d1086* strains was confirmed by PCR using the d1086-2F-3/d1086-2R-2 primer set. Transformation was performed using a previously described standard method (Yang *et al.*, 2015). The transformants were plated onto agar plates containing pravastatin. Pravastatin-resistant colonies were cultured in liquid medium without pravastatin for 96‍ ‍h and then plated on agar plates without pravastatin to isolate the recombinants in which the target gene was replaced with the corresponding disrupted gene. Gene disruption was confirmed by a PCR ana­lysis.

The Δ*c0507*Δ*d1086*(pJ*c0507*) and Δ*c0507*Δ*d1086*(pJ*d1086*)‍ ‍complemented strains were constructed by transforming pJ*c0507* and pJ*d1086*, respectively, into the Δ*c0507*Δ*d1086* strain.

### Extraction and high-performance liquid chromatography (HPLC) and mass spectrometric ana­lyses of total carotenoids from *Ha. japonica*

Wild-type and modified *Ha. japonica* strains were pre-cultured at 37°C. The pre-inoculum (1‍ ‍mL) was then transferred to a 500-mL Erlenmeyer flask containing 100‍ ‍mL of liquid medium and cultured at 37°C for 240‍ ‍h in the dark. Cells were harvested by centrifugation (4,400×*g*, 4°C, 20‍ ‍min), disrupted by sonication in acetone/methanol [7:2 (v/v)], and centrifuged again (840×*g*, 4°C, 5‍ ‍min). Pigments were re-extracted from the pellets. The combined supernatants were evaporated to dryness under a vacuum. Dried carotenoid extracts were used for HPLC and purified carotenoids for mass spectrometric ana­lyses as previously described (Yang *et al.*, 2015).

### DNA sequence accession numbers

The DNA sequence data of *d1086* and *c0507* have been deposited in the DNA Data Bank of Japan, European Molecular Biology Laboratory, and GenBank nucleotide sequence databases. GenBank accession numbers for *d1086* and *c0507* are LC331100 and LC008542, respectively.

## Results

### Phytoene desaturase gene candidates in the *Ha. japonica* genome

By using the Basic Local Align Search Tool, two candidate genes encoding the unidentified phytoene desaturases, *c0507* and *d1086*, were found from the *Ha. japonica* genome (Nakamura *et al.*, 2011). Although C0507 was previously identified as carotenoid 3,4-desatirase (CrtD) (Yang *et al.*, 2015), the enzyme showed 30% identity and E-values of 6e-70 at the amino acid level with *Pantoea ananatis* CrtI (*Pa*CrtI), suggesting its role as a bifunctional enzyme of both 3,4-desaturase (CrtD) and phytoene desaturase (CrtI). Furthermore, D1086 exhibited 27% identity and E-values of 9e-52 with *Pa*CrtI and 60% homology with C0507, suggesting its role also as a bifunctional enzyme.

### Analysis of Δ*d1086* and Δ*c0507* strains

We constructed a *d1086* gene disruptant Δ*d1086* strain. The Δ*d1086* strain showed the same growth rate as the wild-type strain. Carotenoids of the wild-type and Δ*d1086* strains were analyzed by HPLC. The wild-type strain exhibited five carotenoid peaks on HPLC for BR (Fig. 3A, peak 1), MABR (peak 2), BABR (peak 3), IDR (peak 4), and lycopene (peak 5) (Table 3). BR was the major carotenoid in the Δ*d1086* strain. Moreover, its carotenoid composition and HPLC elution profile were similar to those of the wild-type strain. Therefore, the Δ*d1086* strain, similar to the wild-type strain, exhibited both phytoene desaturase (CrtI) and 3,4-desaturase (CrtD) activities. C0507, which acts as CrtD (Yang *et al.*, 2015), may also function as CrtI. Therefore, we redefined C0507 as a bifunctional phytoene desaturase and 3,4-desaturase, CrtI/CrtD(C0507). However, the specific functions of D1086 remain unclear.

We used the previously constructed *c0507* gene disruptant strain of *Ha. japonica*, the Δ*c0507* strain (Yang *et al.*, 2015). The Δ*c0507* strain did not accumulate phytoene, but accumulated tetrahydro-bisanhydrobacterioruberin (TH-BABR) and dihydro-isopentenyldehydrorhodopin (DH-IDR) (Fig. 3B and Table 3), indicating that it exhibited CrtI, but not CrtD activity. Therefore, in the Δ*c0507* strain, TH-BABR and DH-IDR were not desaturated, leading to their accumulation. Furthermore, unlike C0507, D1086 functioned as CrtI.

### Analysis of the Δ*c0507*Δ*d1086* strain

A double-disruptant of both the *c0507* and *d1086* genes, the Δ*c0507*Δ*d1086* strain, was also constructed. The cell pellet of the Δ*c0507*Δ*d1086* strain was colorless (Fig. 3C). The carotenoids of this strain were analyzed by HPLC, and only one carotenoid was detected (Fig. 3C, peak 1’’). Based on its HPLC retention time, absorption spectrum, and mole­cular mass of 544, the carotenoid was identified as phytoene (Table 3). Therefore, the Δ*c0507*Δ*d1086* strain did not exhibit CrtI activity, suggesting that C0507 and D1086 cooperatively catalyzed phytoene desaturation as CrtIs.

### Complementation study on the Δ*c0507*Δ*d1086* strain

An *in vivo* complementation study was performed using the Δ*c0507*Δ*d1086* strain. The Δ*c0507*Δ*d1086* strain was independently transformed using *c0507* and *d1086* gene expression plasmids (pJ*c0507* and pJ*d1086*, respectively) to yield the Δ*c0507*Δ*d1086*(pJ*c0507*) and Δ*c0507*Δ*d1086*(pJ*d1086*) strains, respectively.

The expression of C0507 in the Δ*c0507*Δ*d1086* strain complemented BR biosynthesis (Fig. 3D and Table 3). In addition to BR, BABR was detected as the primary product. In the Δ*c0507* strain, the transcription levels of both the *c0506* and *c0505* genes were reduced, indicating that the disruption of the *c0507* gene affected the expression of the downstream *c0506* and *c0505* genes (Yang *et al.*, 2015). The Δ*c0507*Δ*d1086* strain was then constructed by introducing the disrupted *d1086* gene using the Δ*c0507* strain as the host. The transcription levels of the *c0506* and *c0505* genes were lower in the Δ*c0507*Δ*d1086* and Δ*c0507*Δ*d1086*(pJ*c0507*) strains than in the wild-type strain, leading to the accumulation of intermediates (MABR, BABR, IDR, and lycopene) and a decrease in the percentage of the final product (BR) in the *c0507*Δ*d1086*(pJ*c0507*) strain.

The expression of D1086 in the Δ*c0507*Δ*d1086* strain also complemented BR biosynthesis (Fig. 3E and Table 3), suggesting that D1086 also functioned as CrtD. This result indicates that D1086 participated in the bifunctional conversion desaturation of phytoene to BR, similar to CrtI and CrtD. Therefore, we defined D1086 as a bifunctional phytoene desaturase and 3,4-desaturase, CrtI/CrtD(D1086), similar to C0507. The Δ*c0507*Δ*d1086*(pJ*d1086*) strain synthesized BR; however, the major carotenoid was BABR. Similar to the Δ*c0507*Δ*d1086*(pJ*c0507*) strain, the transcription levels of the *c0506* and *c0505* genes were low, leading to the accumulation of intermediates (MABR, BABR, IDR, and lycopene) and a decrease in the percentage of the final product (BR) in the *c0507*Δ*d1086*(pJ*d1086*) strain.

### Transcriptional ana­lysis of *c0507* and *d1086*

C0507 and D1086 both act as bifunctional CrtI/CrtDs. As shown above, the Δ*c0507* strain accumulated TH-BABR and DH-IDR, whereas the Δ*d1086* strain produced BR similar to the wild-type strain. This discrepancy may be due to differences in the transcription levels of the *c0507* and *d1086* genes in *Ha. japonica*. To verify this, the transcription levels of both genes in the wild-type strain were assessed by RT-PCR. The transcription level of the *d1086* gene was markedly lower than that of the *c0507* gene; however, the *d1086* and *c0507* genes were both transcribed (Fig. 4). This result indicates that the transcription level of the *d1086* gene was extremely low in the Δ*c0507* strain and also that the amount of D1086 was not sufficient to catalyze the desaturation of phytoene and the formation of double bonds at C-3,4 and C-3′,4′ in lycopene derivatives. Therefore, D1086 only exhibited phytoene desaturase (CrtI) activity in the Δ*c0507* strain. In complementation experiments, unlike the Δ*c0507* strain, the Δ*c0507*Δ*d1086*(pJ*d1086*) strain produced BR without the accumulation of intermediates, such as TH-BABR and DH-IDR. The pJ*c0507* and pJ*d1086* expression plasmids contained a powerful promoter of the *Ha. japonica csg* gene. Therefore, the transcription level of the *d1086* gene was higher in the Δ*c0507*Δ*d1086*(pJ*d1086*) strain than in the wild-type strain, and the overexpression of D1086 led to BR production in the Δ*c0507*Δ*d1086*(pJ*d1086*) strain

## Discussion

In the present study, we identified two enzymes with bifunctional phytoene desaturase (CrtI) and 3,4-desaturase (CrtD) activities in the extremely halophilic archaeon, *Ha. japonica*. The first phytoene desaturase (CrtI) was C0507, previously identified as 3,4-desaturase (CrtD) (Yang *et al.*, 2015). The second phytoene desaturase (CrtI) was D1086; similar to C0507, it also functioned as a 3,4-desaturase (CrtD). C0507 and D1086 complemented each other during carotenoid biosynthesis in *Ha. japonica*. To the best of our knowledge, this study marks the first identification of two functional CrtIs in archaea. Furthermore, functional enzymes exhibiting both phytoene desaturase and 3,4-desaturase activities have not been previously reported, suggesting the novelty of the enzymes identified herein.

**Fig. 1.**
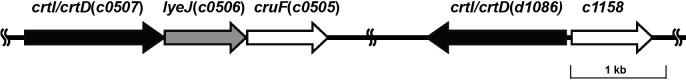
Organization of gene clusters for BR and retinal biosynthesis in *Haloarcula japonica*. The BR biosynthesis gene cluster consists of the *crtI/crtD*(*c0507*), *lyeJ*(*c0506*), and *cruF*(*c0505*) genes, whereas the retinal biosynthesis gene cluster consists of the *crtI/crtD*(*d1086*) and *c1158* genes. Notably, the *c1158* gene is homologous to the *brp* gene of *Ha. japonica*. Moreover, the *c1219* gene, homologous to the *crtY* gene of *Ha. japonica*, is located in a distinct genomic region.

**Fig. 3.**
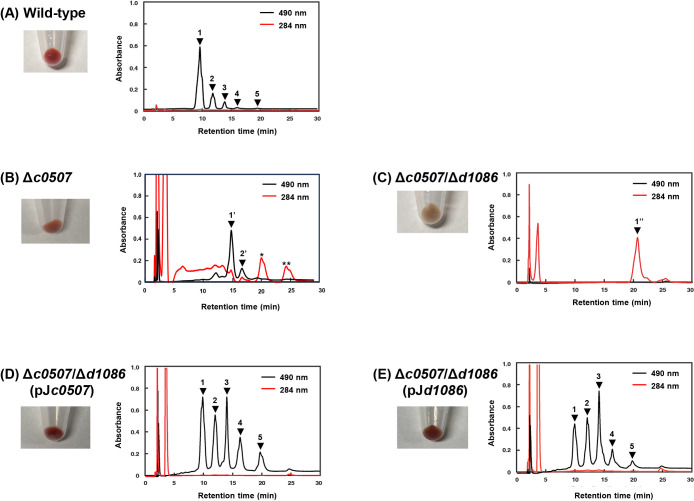
Cell pellets of wild-type, disruptant, and complemented strains, and HPLC elution profiles of their carotenoids. (A) Wild-type strain. (B) Δ*c0507* strain. (C) Δ*c0507*Δ*d1086* strain. (D) Δ*c0507*Δ*d1086*(pJ*c0507*) strain., (E) Δ*c0507*Δ*d1086*(pJ*d1086*) strain. Peak 1, BR; peak 2, MABR; peak 3, BABR; peak 4, IDR; peak 5, lycopene; peak 1’, TH-BABR; peak 2’, DH-IDR; peak 1’’, phytoene. The absorption spectra of the two peaks indicated by asterisks (* and **) differ from those of phytoene. The eluent was methanol-water [9:1 (v/v)] for the first 10‍ ‍min and was then changed to 100% methanol (1.5‍ ‍mL‍ ‍min^–1^). All carotenoids, except for phytoene, were detected at 490‍ ‍nm, whereas phytoene was detected at 284‍ ‍nm.

**Fig. 4.**
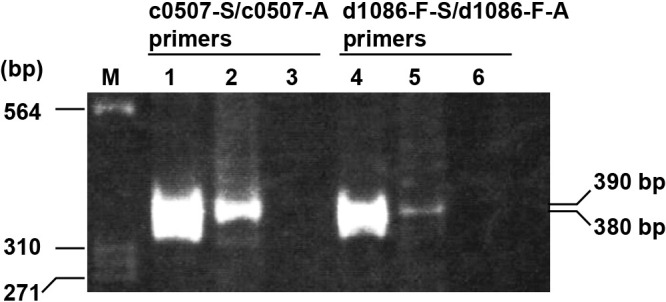
Agarose gel electrophoresis of RT-PCR products using c0507-S/c0507-A and d1086-S/d1086-A primers. Lanes 1 and 4: positive control reaction products using *Haloarcula japonica* genomic DNA as the template; lanes 2 and 5: RT-PCR products with total RNA as the template; lanes 3 and 6: negative control reaction products without reverse transcription.

**Fig. 5.**
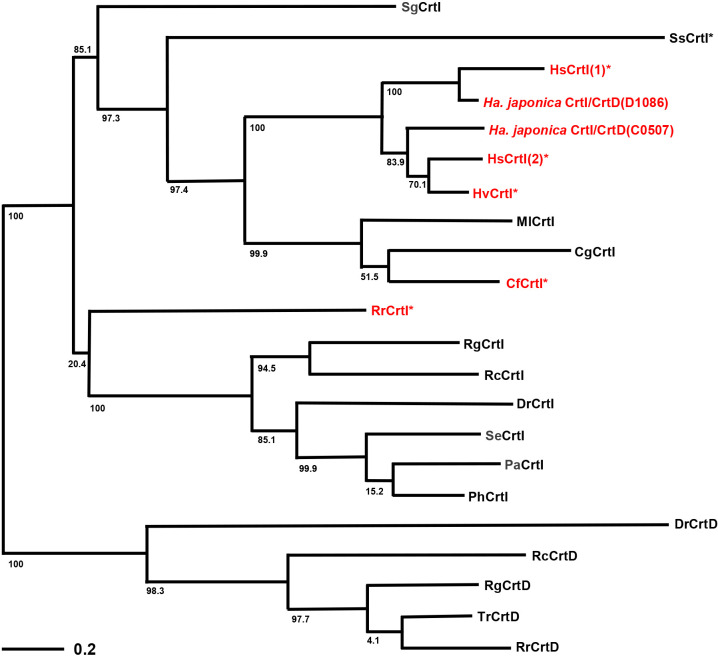
Phylogenetic tree of CrtI and CrtD homologues. The abbreviations of enzymes and the accession numbers of genomes are as follows: SgCrtI, *Streptomyces griseus* CrtI (AAA91950.1); SsCrtI, *Saccharolobus solfataricus* P2 CrtI (AAK43016.1); HsCrtI(1) and HsCrtI(2), *Halobacterium salinarum* NRC-1 CrtIs (AAG19932.1, and DAC78712.1, respectively); HvCrtI, *Haloferax volcanii* DS2 CrtI (ADE03359.1); MlCrtI, *Micrococcus luteus* CrtI (QZY83930.1); CgCrtI, *Corynebacterium glutamicum* CrtI (AAK64299.1); CfCrtI, *Cutobacterium flaccumfaciens* CrtI (WP_111022696.1); RrCrtI, *Rubrobacter radiotolerans* CrtI (AHY46015.1); RgCrtI, *Rubrivivax gelatinosus* CrtI (TCP03938.1); RcCrtI, *Rhodobacter capsulatus* CrtI (WER10061.1); DrCrtI, *Deinococcus radiodurans* CrtI (WP_010887507.1); SeCrtI, *Sphingomonas elodea* ATCC 31461 CrtI (AEP37354.1); PaCrtI, *Pantoea ananatis* CrtI (PZD69089.1); PhCrtI, *Paracoccus haeundaensis* CrtI (AAY28420.1); DrCrtD, *Deinococcus radiodurans* CrtD (AAF11796.1); RcCrtD, *Rhodobacter capsulatus* (CAA77544.1); RgCrtD, *Rubrivivax gelatinosus* CrtD (BAL96698.1); TrCrtD, *Thiocapsa roseopersicina* CrtD (AAP59036.1). RrCrtD, *Rhodospirillum rubrum* CrtD (AAN75037.1). Proteins annotated as Crts with unknown functions are indicated by asterisks (*). CrtIs from microorganisms synthesizing BR and/or BR analogs are indicated in red. Sequences were aligned using CLUSTALW, and a phylogenetic tree was constructed using the neighbor-joining (NJ) method. Branch length labels are illustrated using bars, and each bootstrap value is annotated with a numerical label.

Fig. 5 shows the evolutionary phylogenetic tree of the previously identified functional CrtIs and CrtDs as well as‍ ‍*Ha. japonica* CrtI/CrtD(C0507) and CrtI/CrtD(D1086) reported in this study. The tree includes sequences annotated as CrtI for BR- and/or BR analog-producing microbes. CrtIs and CrtDs are desaturases that share significant homology with each other. A phylogenetic tree ana­lysis revealed that‍ ‍CrtDs originated from CrtIs via gene duplication at an early stage, resulting in the divergence of CrtIs and CrtDs with unique functions. The tree shows that CrtI/CrtD(C0507) and CrtI/CrtD(D1086) were closely related members of the CrtI family. The *Ha. japonica crtI/crtD*(*d1086*) gene was located upstream of *c1158*, which is homologous to the *Hb. salinarum brp* gene, in opposite directions (Fig. 1). Consequently, CrtI/CrtD(D1086) may be involved in retinal biosynthesis and CrtI/CrtD(C0507) in BR biosynthesis. In BR biosynthesis, not only CrtI activity, but also CrtD activity is essential. Therefore, the ancestral CrtI(C0507) in the *Ha. japonica* genome may have underwent the expansion of its substrate specificity during evolution, subsequently acquiring CrtD activity and evolving into bifunctional CrtI/CrtD(C0507). As shown in Fig. 5, *Ha. japonica* was slower to acquire CrtI/CrtD(D1086) than CrtI/CrtD(C0507). Therefore, *Ha. japonica* acquired the retinal biosynthetic pathway via gene duplication of the already acquired BR biosynthetic genes. Since CrtI(C0507) evolved into bifunctional CrtI/CrtD(C0507), CrtI/CrtD(D1086) inevitably exhibited dual functionality. *Hb. salinarum* also produces both BR and retinal and possesses two CrtIs [HsCrtI(1) and HsCrtI(2)]. Closely related *Holoferax volcanii* produces BR, but not retinal, with only one CrtI (HvCrtI). CrtI and CrtD activities are both essential for BR biosynthesis, and CrtIs in these archaea may also be bifunctional enzymes. Microbes possessing bifunctional CrtI(s) are not necessarily limited to extremely halophilic archaea, such as *Ha. japonica*. Two bacterial CrtIs, RrCrtI from *Rubrobacter radiotolerans* and CfCrt from *Cutobacterium flaccumfaciens*, may also act as bifunctional enzymes because *R. radiotolerans* and *C. flaccumfaciens* both only possess one copy of CrtI and produce BR.

To examine the functions of CrtI/CrtD(C0507) and CrtI/CrtD(D1086) in more detail, we expressed *crtI/crtD*(*c0507*) and *crtI/crtD*(*d1086*) in *E. coli* BL21(DE3) cells containing pAC-EB1 (Furubayashi *et al.*, 2015) to facilitate phytoene production using the T7 promoter. Carotenoids were not produced, except for phytoene, in *E. coli*; however, the expression of CrtI/CrtD(C0507) and CrtI/CrtD(D1086) was detected by Western blotting of His-tagged CrtI/CrtD(C0507) and CrtI/CrtD(D1086) probed with an anti-His-tag antibody (data not shown). Since enzymes from extremely halophilic archaea generally require high salt concentrations for their activity and stability, *E. coli*-produced CrtI/CrtD(C0507) and CrtI/CrtD(D1086) in the present study may not function under physiological conditions. Further studies are needed to characterize these enzymes at the mole­cular level.

## Citation

Yatsunami, R., Ando, A., Miyoko, N., Yang, Y., Takaichi, S., and Nakamura, S. (2024) Two Distinct Enzymes Have Both Phytoene Desaturase and 3,4-Desaturase Activities Involved in Carotenoid Biosynthesis by the Extremely Halophilic Archaeon *Haloarcula japonica*. *Microbes Environ ***39**: ME24004.

https://doi.org/10.1264/jsme2.ME24004

## Figures and Tables

**Fig. 2.**
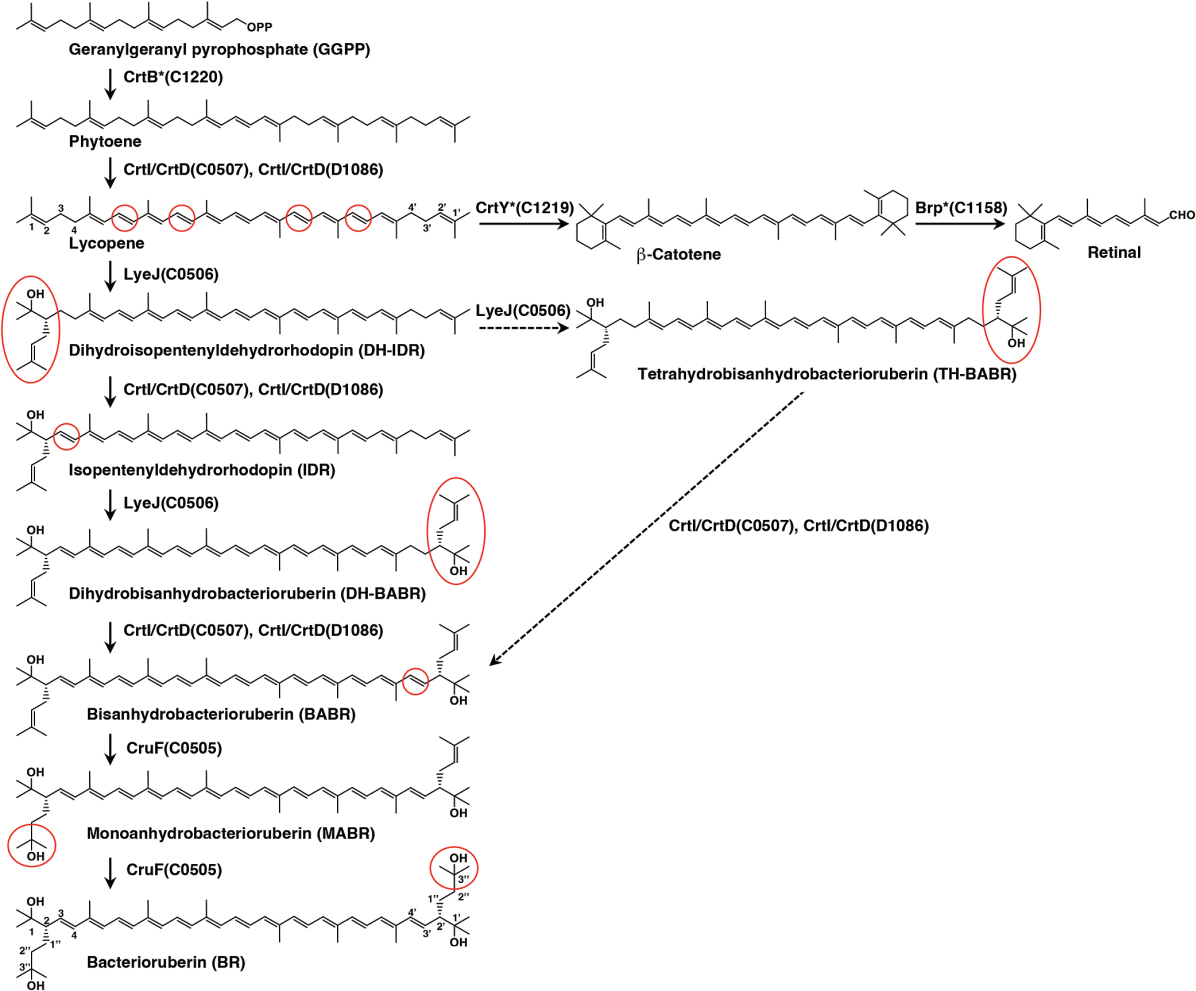
Main steps in BR and retinal biosynthetic pathways of *Haloarcula japonica*. Solid arrows indicate the main BR biosynthetic steps. Dashed arrows indicate other possible BR biosynthetic steps. Among the intermediates from phytoene to BR, the locations of structural changes in each step are circled in red. Asterisks indicate unidentified BR and retinal biosynthetic enzymes.

**Table 1.** Microbial strains and plasmids used in this study

Strain or plasmid	Relevant property	Source or reference
Strains		
*E. coli* JM109	Host used for gene cloning	Laboratory stock
*E. coli* JM110	Host used for preparing plasmids that are free of Dam and Dcm methylation	Laboratory stock
*Ha. japonica*	Wild-type strain of *Ha. japonica* (JCM 7785^T^)	Horikoshi *et al.*, 1993
*Ha. japonica* Δ*d1086*	*d1086* gene disruptant of *Ha. japonica*	This work
*Ha. japonica* Δ*c0507*	*c0507* gene disruptant of *Ha. japonica*	Yang *et al.*, 2015
*Ha. japonica* Δ*c0507*Δ*d1086*	Double disruptant of both the *c0507* and *d1086* genes of *Ha. japonica*	This work
Plasmids		
pUC119	*E. coli* cloning vector; Ap^r^	Laboratory stock
pWL102	*E. coli*-extremely halophilic archaea shuttle vector; Ap^r^, Mev^r^	Lam and Doolittle, 1989
pJFZ33	pWL102 derivative carrying the *Ha. japonica csg* promoter and *ftsZ2* structural gene; Ap^r^, Mev^r^	Ozawa *et al.*, 2005
pJ*c0507*	pJFZ33 derivative in which the *ftsZ2* structural gene is replaced by the *c0507* structural gene; Ap^r^, Mev^r^	Yang *et al.*, 2015
pCRTI2	pUC119 derivative screened from the *Pst*I sub-genomic library; Ap^r^	This work
pWL102(Δ*ori*)-Δ*d1086*	pWL102 derivative containing a disrupted fragment of the *d1086* structural gene; Ap^r^, Mev^r^	This work
pUC119-*d1086*	pUC119 derivative containing the *d1086* structural gene; Ap^r^	This work
pJ*d1086*	pJFZ33 derivative in which the *ftsZ2* structural gene is replaced by the *d1086* structural gene; Ap^r^, Mev^r^	This work

Ap^r^, ampicillin resistance; Mev^r^, mevinoline resistance.

**Table 2.** Sequences of the primers used in this study

Primer	Sequence
c0507-S	5′-GGTTGGCCTCCAGCTCATTG-3′
c0507-A	5′-CTCGTGGTCCGGCAGGAGTTC-3′
d1086-F-S	5′-AACATGAACGTGTTCTACCC-3′
d1086-F-A	5′-AATATCTGCTCGAAGTGGTC-3′
d1086-S3	5′-GATTCCTATGGTGGGTATGCG-3′
d1086-A3	5′-GGGATCCTCATTAGTGGTGGTGGTGGTGGTGGTCCGCTTTCGAGGCCGAGC-3′
d1086-2F-3	5′-GTTCGAATCCTACGAGGATG-3′
d1086-2R-2	5′-GACGACCTCGCGAAGGAGTTC-3′

**Table 3.** Characteristics of carotenoids extracted from wild-type, disruptant, and complementation strains

Strain	Peak no.	Retention time (min)	λ_max_ (nm) in HPLC eluent	Number of conjugated double bonds	Carotenoid
Wild-type	1	9.6	469, 492, 525	13	BR
	2	11.8	469, 491, 522	13	MABR
	3	13.8	467, 490, 521	13	BABR
	4	16.0	453, 479, 509	12	IDR
	5	19.4	442, 468, 499	11	Lycopene
Δ*c0507*	1’	14.8	441, 466, 498	11	TH-BABR
	2’	16.7	440, 465, 495	11	DH-IDR
Δ*c0507*Δ*d1086*	1’’	21.3	273, 284, 297	3	Phytoene
Δ*c0507*Δ*d1086*(pJ*c0507*)	1	9.8	461, 490, 520	13	BR
	2	11.9	460, 489, 520	13	MABR
	3	13.9	460, 489, 521	13	BABR
	4	16.2	452, 480, 509	12	IDR
	5	19.6	441, 468, 498	11	Lycopene
Δ*c0507*Δ*d1086* (pJ*d1086*)	1	9.8	460, 490, 522	13	BR
	2	11.9	462, 490, 523	13	MABR
	3	13.9	460, 489, 521	13	BABR
	4	16.2	452, 480, 511	12	IDR
	5	19.5	440, 468, 497	11	Lycopene

Carotenoids were analyzed using HPLC equipped with a μBondapak C_18_ column (3.9×300‍ ‍mm; Waters) and eluted with methanol/water [9:1 (v/v)] for the first 10‍ ‍min and then with 100% methanol (1.5‍ ‍mL‍ ‍min^–1^). The absorption spectra of carotenoids were recorded with a photodiode-array detector attached to the HPLC apparatus (Fig. 3).

## References

[B1] Dummer, A.M., Bonsall, J.C., Cihla, J.B., Lawry, S.M., Johnson, G.C., and Peck, R.F. (2011) Bacterioopsin-mediated regulation of bacterioruberin biosynthesis in *Halobacterium salinarum*. J Bacteriol 193: 5658–5667. 21840984 10.1128/JB.05376-11 PMC3187228

[B2] Dundas, I.D., and Larsen, H. (1963) A study on the killing by light of photosensitized cells of *Halobacterium salinarium*. Arch Mikrobiol 46: 19–28. 14054143 10.1007/BF00406383

[B3] Furubayashi, M., Ikezumi, M., Takaichi, S., Maoka, T., Hemmi, H., Ogawa, T., et al. (2015) A highly selective biosynthetic pathway to non-natural C_50_ carotenoids assembled from moderately selective enzymes. Nat Commun 6: 7534. 26168783 10.1038/ncomms8534 PMC4510654

[B4] Holt, N.E., Zigmantas, D., Valkunas, L., Li, X.P., Niyogi, K.K., and Fleming, G.R. (2005) Carotenoid cation formation and the regulation of photosynthetic light harvesting. Science 307: 433–436. 15662017 10.1126/science.1105833

[B5] Horikoshi, K., Aono, R., and Nakamura, S. (1993) The triangular halophilic archaebacterium *Haloarcula japonica* strain TR-1. Experientia 49: 497–502.

[B6] Lam, W.L., and Doolittle, W.F. (1989) Shuttle vectors for the archaebacterium *Halobacterium volcanii*. Proc Natl Acad Sci U S A 86: 5478–5482. 2748598 10.1073/pnas.86.14.5478 PMC297646

[B7] Lazrak, T., Wolff, G., Albrecht, A.M., Nakatani, Y., Ourisson, G., and Kates, M. (1988) Bacterioruberins reinforce reconstituted *Halobacterium* lipid membranes. Biochim Biophys Acta Biomembr 939: 160–162.

[B8] Miller, N.J., Sampson, J., Candeias, L.P., Brameley, P.M., and Rice-Evans, C.A. (1996) Antioxidant activities of carotenes and xanthophylls. FEBS Lett 384: 240–242. 8617362 10.1016/0014-5793(96)00323-7

[B9] Naguib, Y.M. (2000) Antioxidant activities of astaxanthin and related carotenoids. J Agric Food Chem 48: 1150–1154. 10775364 10.1021/jf991106k

[B10] Nakamura, S., Nakasone, K., and Takashina, T. (2011) Genetics and genomics of triangular disc-shaped halophilic archaeon *Haloarcula japonica* TR-1. In *Extremophiles Handbook*, vol 2. Horikoshi, K., Antranikian, G., Bull, A.T., Robb. F.T., and Stetter, K.O. (eds). Heidelberg: Springer, pp. 364–381.

[B11] Oesterhelt, D., and Stoeckenius, W. (1971) Rhodopsin-like protein from the purple membrane of *Halobacterium halobium*. Nat New Biol 233: 149–152. 4940442 10.1038/newbio233149a0

[B12] Onodera, M., Yatsunami, R., Tsukimura, W., Fukui, T., Nakasone, K., Takashina, T., et al. (2013) Gene ana­lysis, expression, and characterization of an intracellular amylase from the extremely halophilic archaeon *Haloarcula japonica*. Biosci Biotechnol Biochem 77: 281–288. 23391916 10.1271/bbb.120693

[B13] Ozawa, K., Harashina, T., Yatsunami, R., and Nakamura, S. (2005) Gene cloning, expression and partial characterization of cell division protein FtsZ1 from extremely halophilic archaeon *Haloarcula japonica* strain TR-1. Extremophiles 9: 281–288. 15844012 10.1007/s00792-005-0443-6

[B14] Peck, R.F., Echavarri-Erasun, C., Johnson, E.A., Ng, W.V., Kennedy, S.P., Hood, L., et al. (2001) *Brp* and *blh* are required for synthesis of the retinal cofactor of bacteriorhodopsin in *Halobacterium salinarum.* J Biol Chem 276: 5739–5744. 11092896 10.1074/jbc.M009492200

[B15] Peck, R.F., Johonson, E.A., and Krebs, M.P. (2002) Identification of a lycopene β-cyclase required for bacteriorhodopsin biogenesis in the archaeon *Halobacterium salinarum*. J Bacteriol 184: 2889–2897. 12003928 10.1128/JB.184.11.2889-2897.2002 PMC135044

[B16] Sambrook, J., Fritsch, E.F., and Maniatis, T. (1989) *Molecular Cloning: A Laboratory Manual.* Cold Spring Harbor, NY: Cold Springer Harbor Laboratory Press.

[B17] Sandmann, G. (2021) Diversity and origin of carotenoid biosynthesis: its history of coevolution towards plant photosynthesis. New Phytol 232: 479–493. 34324713 10.1111/nph.17655

[B18] Shahmohammadi, H.R., Asgarani, E., Terato, H., Saito, T., Ohyama, Y., Gekko, K., et al. (1998) Protective roles of bacterioruberin and intracellular KCl in the resistance of *Halobacterium salinarium* against DNA damaging agents. J Radiat Res 39: 251–262. 10196780 10.1269/jrr.39.251

[B19] Shimada, K., and Takaichi, S. (eds). (2024) *Anoxygenic Phototrophic Bacteria*. Cambridge, MA: Academic Press.

[B20] Takaichi, S., and Mochimaru, M. (2007) Carotenoids and carotenogenesis in cyanobacteria: Unique ketocarotenids and carotenoid glycosides. Cell Mol Life Sci 64: 2607–2619. 17643187 10.1007/s00018-007-7190-z PMC11136355

[B21] Takaichi, S. (2013) Tetraterpenes: Carotenoids. In *Natural Products: Phytochemistry, Botany and Metabolism of Alkaloids, Phenolics and Terpenes.* Ramawat, K.G., and Mérillon, J.-M. (eds). New York, NY: Springer, pp. 3251–3283.

[B22] Yang, Y., Yatsunami, R., Ando, A., Miyoko, N., Fukui, T., Takaichi, S., et al. (2015) Complete biosynthetic pathway of the C_50_ carotenoid bacterioruberin from lycopene in the extremely halophilic archaeon *Haloarcula japonica*. J Bacteriol 197: 1614–1623. 25712483 10.1128/JB.02523-14 PMC4403650

[B23] Yatsunami, R., Kawakami, T., Ohtani, H., and Nakamura, S. (1999) Transcriptional regulation of cruxrhodopsin gene from extremely halophilic archaeon *Haloarcula japonica* strain TR-1. Nucleic Acids Symp Ser 42: 73–74. 10.1093/nass/42.1.73 10780385

[B24] Yatsunami, R., Iwamoto, M., Ito, S., and Nakamura, S. (2003) A *brp* homolog of *Haloarcula japonica*: gene cloning and transcriptional ana­lysis. Nucleic Acids Res Suppl 3: 267–268. 10.1093/nass/3.1.267 14510483

[B25] Yatsunami, R., Ando, A., Yang, Y., Takaichi, S., Kohno, M., Matsumura, Y., et al. (2014) Identification of carotenoids from the extremely halophilic archaeon *Haloarcula japonica*. Front Microbiol 5: 100–105. 24672517 10.3389/fmicb.2014.00100 PMC3956123

